# The British Medical Association at Oxford

**Published:** 1904-08-06

**Authors:** 


### The British Medical Association at Oxford.

The seventy-second annual general meeting of the British Medical Association was opened on the 26th ult. in the Examination Schools, Oxford. The chair was occupied by last year's president, Dr. T. D. Griffiths. After briefly alluding to the death of Sir John Simon, who, he said, "had saved far more lives during the last 50 years than all the armies of the world had destroyed during the same period," Dr. Griffiths gave his valedictory address, and vacated the chair, which was then occupied by his successor, Dr. Collier, the president for the current year.

### President's Address.

Dr. Collier chose for the subject of his address the "Growth and Development of the Oxford Medical School." He pointed out that originally the Faculty of Medicine had been placed on a par with those of Divinity and Law, and the provisions for teaching and graduating in all these faculties had been made alike. Then, however, there followed a decline in the prosperity of the Medical Faculty, due in the main to the successful rivalry of the College of Physicians in London, until in a short time little was left to the University beyond the function of giving a preliminary training to the few who could avail themselves of it. The revival commenced in 1878 by a correspondence in the medical press, which culminated in the following year in a memorial sent by the British Medical Association to the Hebdomadal Council, to the House of Commons, and to the then recently-appointed University Commission. This memorial pointed out the advantages likely to follow the establishment of a medical school at Oxford, and urged the immediate constitution of a thorough medical curriculum, on the same basis as the medical schools of other English centres, in the following subjects at least:—(1) Human Anatomy; (2) Physiology; (3) General Pathology; (4) Materia Medica; (5) Clinical Medicine and Surgery for Beginners; (6) State Medicine, including Medical Jurisprudence and Public Health.

### The First Step.

In November 1882 the first step was taken towards the establishment of medical education in Oxford on modern lines, by the appointment of Professor Burdon Sanderson to the newly founded Chair of Human Physiology and Histology at Oxford. Having elected a Professor of Physiology, it became necessary to build laboratories and class-rooms for teaching purposes, and on June 5th, 1883, in the face of keen opposition, a sum of £10,000 was granted for this purpose by Convocation, with the small majority of three votes. In 1885 a similar grant was made for the same object.

### Recreation of the Faculty of Medicine.

In this year the Faculty of Medicine, which had been merged in natural science, was recreated, and Mr. Arthur Thomson was elected Lecturer in Anatomy. When he commenced his work in Oxford, Mr. Thomson found himself with three students and a small wooden shed in which to teach anatomy. A sum of money was immediately collected by members of the university interested in the development of the school, and a small temporary lecture-room and a dissecting-room were built. By 1891 the lecturer's class had grown from 3 to 67. A sum of £7,000 was now asked for and obtained from the university to provide a permanent home for the teaching of human anatomy, and the present building was erected. In a year or two, largely owing to the success of Mr. Thomson as a teacher, the lectureship was converted into a professorship.

In 1886 a most important statute was passed, by which students in natural science were exempted from the first public examination in classics, known as moderations. This measure enabled students of medicine, after passing their preliminary examination, known as responsions, which might be passed before coming into residence, to devote their first year in Oxford to the study of the preliminary subjects in natural science, and so another and most important barrier to the establishment of a medical school disappeared. In 1891 Sir Henry Acland, who had always interested himself in the study of pathology and bacteriology, inaugurated a new department in bacteriology and appointed, first, Dr. Menge, of Munich, to take charge of it, and, later, in turn, Dr. Bertram Hunt and Dr. Richie.

In 1896 the Regius Professor, Sir John Burdon Sanderson, in an open letter to the President of Magdalen, pointed out that the time had come when Oxford medical students should have the advantage of two new departments—pathology and pharmacology—both well within the scope to which Oxford had restricted itself in the building up of the medical school, and in the following year William John Smith Jerome was appointed Lecturer in Pharmacology and Materia Medica.

In May 1898, in spite of the impoverished condition of the University Chest, Convocation passed without dissent a decree empowering the University to spend £7,500 in erecting new laboratories and lecture-rooms for the joint use of the professors of botany and comparative anatomy, Sir William Anson showing that this expenditure was rendered necessary by the increase in the number of medical students.

In 1899 a generous medical student, Mr. Ewan Frazer, offered the University a sum of £5,000 towards the expenses of building a pathological laboratory. This sum was accepted, the University at the same time agreeing to supplement it with another £5,000, and to make an annual grant of £250 for upkeep. Two years later, in October 1901, the new Pathological Laboratory was opened, and at the opening ceremony Dr. Ritchie, reader in pathology, pointed out:—"That, in addition to giving those who were seeking degrees the opportunities of acquiring the necessary knowledge in pathology, it was the desire of the University to provide opportunities for those who wished after graduation to return to Oxford to prosecute researches in pathology and bacteriology, and to this end there had been provided a special room for experimental pathology. In view of the fact that much of the pathological work of the future must be in the direction of the chemical examination of the products of disease processes, a chemical research laboratory had also been provided."

In 1901 the Drapers' Company presented to the University the magnificent medical library, which would ever remain as a memento of their generosity; and in 1902 Mrs. Ogilvie gave to the University a sum of £7,000 for the purpose of creating a Readership in Ophthalmology, to be held by the senior surgeon to the Oxford Eye Hospital.

### The Present State of the Oxford Medical School.

It could now be judged to what extent the desires expressed by the British Medical Association in 1879 had been fulfilled. It had asked that at least human anatomy, physiology and general pathology, and materia medica might be properly taught. Now, for many years past adequate provision and suitable accommodation and apparatus had been provided for the teaching of these subjects, and they were being taught by professors, readers, and lecturers whose ability and fitness for their important duties could not be questioned. The Association had desired that provision should be made for the elementary teaching of clinical medicine and surgery. In 1883 the existing Litchfield Clinical Professorship was split up into a Litchfield Clinical Lectureship in Medicine and one in Surgery, and from that date to the present students had been taught in the wards of the Radcliffe Infirmary the bare outlines of clinical diagnosis in medicine and surgery, and were thus fitted to take immediate advantage of the most advanced teaching and the excellent clinical material which were found at the large metropolitan hospitals.

### The Influence of the School.

Dr. Collier believed that the benefits accruing from the revival of the Oxford medical school were not confined to the University. To his mind the greatest advantage to be noted had been the influence exerted by the university student on the other students of the great metropolitan schools. He asked for an explanation of the fact that while the medical student of a quarter of a century ago undoubtedly bore a very bad character in the eyes of the public, he now no longer does so. The change was largely due, he thought, to the influence of the university graduates, who were older men, had long passed their first experience of freedom from school discipline, had a more settled purpose in life, and had a clearer and better idea of what was so difficult to define, but so easy to recognise—good and bad form.

### ADDRESS IN MEDICINE.

Sir William Church, who gave the address in medicine, briefly reviewed the progress of medicine from 1868 to the present time. The date was chosen both because it represented the year in which the British Medical Association had last met at Oxford and because it marked the beginning of many important advances in medicine. In the previous year Lister had read at Dublin his paper on the "Antiseptic Principle in the Practice of Surgery." It was at this time also that the infectivity of tuberculous matter began to be generally recognised; and since that date great advances had been made in bacteriology and in the subject of immunisation and the phenomena presented by the agglutinative, bactericidal, and hæmolytic properties of the blood serum under certain conditions. The investigations which had been so actively carried on recently in these directions had greatly increased our knowledge of the blood and its component parts, and they might, he believed, lead to still further advances in the field of serum-therapeusis—a field which offered a hopeful prospect of providing a way to counteract the bad effects to which specific disease-producing organisms were capable of giving rise in the living body.

### Tropical Medicine.

After alluding to the important physiological discovery of internal secretions, Sir William Church referred to the remarkable progress made in recent years in the field of tropical diseases. Until a few years ago, he remarked, our knowledge of the part played by the animal kingdom in the dissemination and propagation of disease in man had made but little advance. M. Laveran in 1880 first discovered and described the organism of paludism in the blood, and his discovery, like that of the tubercle bacillus by Koch in 1882, formed an epoch in the history of medicine. Already the work done, mainly under the auspices of the Liverpool and London Schools of Tropical Medicine, had begun to bear fruit not merely by increasing our knowledge of malaria and certain other forms of tropical disease, but by having had in the short space that had elapsed a marked influence in diminishing sickness and mortality in places which were formerly so deadly to European life that trade could hardly be maintained with them. Nor was it only in these hot-beds of fever that beneficial results had been obtained, as Professor Boyce's account of anti-malarial measures taken at Ismailia showed. The result of the sanitary work put in action by the authorities of the Suez Canal had been eminently satisfactory. The attacks of malaria in a population consisting of about 1,900 or 2,000 Europeans and 7,000 natives had fallen from 2,089 in 1897 to 209 in 1903, and the mortality had correspondingly lessened. Those highly satisfactory results had been obtained at comparatively small expense, the Canal Company having expended £4,400 in filling up and draining the marshy ground, whilst the special anti-malarial forces—the Drainage and the Petroleum Brigades—had together cost £720 a year, figures which contrasted most strikingly with a previous "expenditure of £15,000 on works designed with the intention of improving the sanitary condition of the town and £13,000 in medicine and medical attendance," without the production of any beneficial results.

### A Plea for a Physical Census.

After briefly outlining the history of sanitary legislation in this country, Sir William Church expressed a hope that the Committee of the Privy Council on the physical disability of recruits, then engaged in framing its report, would urge on the Government the desirability for a physical census of the nation being taken and renewed from time to time, so that standards might be available for future comparisons; and he urged that the physical condition of school children in primary schools should be watched, recorded, and reported on as regularly and carefully as their educational progress. It was only by information of that sort, collected by competent persons and duly weighed by those competent of forming an opinion on the medical aspects of the question that improvement could be hoped for.

### The Codification of Public Health Law.

Sir William Church lamented that the mass of sanitary legislation and the involved nature of it prevented the public and the profession also, from being able to follow it. Legislation bearing on the national health was, he thought, in advance of administration, and it was not so much fresh legislation that was needed as the simplification and codification of the existing law. The administration of the law could not be much in advance of the knowledge and feeling of the public; and while giving full credit to municipal and other sanitary authorities for the good work which had already been done and the efforts they were then making, the medical officers of health throughout the country were, he said, painfully aware how great was the ignorance shown by the mass of the population of the principles on which the public health depended, and how great was the necessity for educating the general public to a proper appreciation of the elemental facts of sanitary science.

### ADDRESS IN SURGERY.

Sir William Macewen chose as the themes for his address in surgery three subjects. The first dealt with the channels through which cerebral infection might occur.

### Localisation of Brain Abscess arising from Primary Pyogenic Cranial Lesions.

Prior to the advent of cerebro-spinal surgery in its later developments, he said, abscess of the brain was regarded solely as of a pyæmic nature, occurring by metastasis, and, owing to the aberrant manner in which abscess formed, its localisation was regarded as impossible, except in the few instances where abscess happened to occur in parts which gave rise to functional manifestations. Even where abscess occurred as secondary to a primary pyogenic cranial lesion, some were inclined to believe that the brain abscess was equally a pyæmic manifestation, and, accordingly, could occur at any part of the brain distant from, and without relation to, the primary lesion. Observation had shown that theory to be untenable, and it was now known that all brain abscesses arising from the foci within the cranium were, if not in direct contact with such foci, at least in contact with the infected path which lay between the primary focus and the abscess. If the primary cranial pyogenic focus were known, it was certain that the abscess would be situated in direct contiguity with the infected structures. The importance of that fact could be seen in the case of cerebral abscess following suppuration in the middle ear, as the pus could be evacuated, whether it was present in the cerebrum or cerebellum, by extension of the aperture made in the mastoid in order to approach the primary focus.

### The Determination of Abscess or Meningitis.

Anatomical features were, he said, secondary determining factors between abscess and meningitis, and in the latter case between localised and generalised lesions. If the access of the pyogenic organisms had been by way of the tegmen or the sigmoid, abscess of the brain or cerebellum was frequent; and if meningitis resulted, it was at least primarily localised, did not immediately involve vital structures, and was often amenable to prompt treatment.

If, on the other hand, the pyogenic organisms had travelled by way of the internal auditory meatus they occasioned leptomeningitis of a serious kind both on account of its being basal and early involving the medulla and respiratory centres, and because of its inaccessibility. Abscess of the brain seldom or never occurred by pyogenic organisms travelling by way of the internal auditory meatus.

### Tuberculosis of the Middle Ear extending to the Brain.

Sir William Macewen stated that he had seen ten cases in which tuberculous meningitis had occurred in association with tuberculosis of the middle ear unaccompanied by perforation of the tympanic membrane, and in the majority of cases without swelling over the mastoid. In five of the cases the infection had spread by the internal auditory meatus. Another mode by which tuberculosis of the middle ear might become a menace to life was by dissemination through the sigmoid sinus to other parts of the body, and he had himself seen a case of acute general tuberculosis in which post-mortem examination showed both mastoids filled with granulation tissue, while the right sinus was penetrated and the aperature was in direct contact with caseating tubercle.

### A Respiratory and Cardiac Reflex induced by Stimulation of the Pudic Nerves.

Having noticed that a deeply anæsthetised patient exhibited signs of spasm of the glottis at the moment when the sphincter ani was stretched, Sir William Macewen was led to make further observations, with the result that he became assured of a definite cardio-respiratory reflex which could be elicited by stimulation of the pudic nerve or the perinæal branch of the fourth sacral, even though the patient were deeply anæsthetised. The effect upon the heart, he stated, was as a rule not nearly so marked as that upon respiration. The further observation was made that when any prior obstruction to respiration existed, such as to induce venosity of the blood, the respiratory centre became more easily affected by the pudic afferent impulses. The reflex phenomena occasioned by stimulation of the pudic nerve might be prevented by local anæsthesia, and Sir William Macewen recommended a combination of this latter with general anæsthesia in order to control the reflex during operations in the pudic area.

### Choice of Material for Ligatures and Sutures.

Sir William Macewen pointed out that in selecting material for ligatures and sutures it was desirable to choose some substance that would undergo absorption in the tissues and he thought it a mistake to regard the possibility of asepticity as the sole requirement. What was wanted was a material which would keep the tissues in contact for a sufficient time to allow them to unite and which would then become absorbed. For these purposes good catgut was one of the best substances available, and in preparing it for use different degrees of resistance could be imparted to it and its rate of absorption in the tissues could be thus regulated. This end could be achieved by immersing the catgut in a mixture of chromic acid and glycerine for varying periods, the degree of hardness produced being proportional to the period of immersion. In this way sutures could be prepared which, before undergoing absorption, would hold aponeurosis or muscle in contact for three weeks, and others, again, could be obtained, suitable for closing skin incisions, which would be absorbed in a week. In the latter case a great advantage was secured in that it was unnecessary, provided the wound remained aseptic, to interfere with it from the date of operation until healing was complete.

